# Crystal structures of PigF, an *O*-methyltransferase involved in the prodigiosin synthetic pathway, reveal an induced-fit substrate-recognition mechanism

**DOI:** 10.1107/S2052252521011696

**Published:** 2022-03-01

**Authors:** Shenshen Qiu, Dongqing Xu, Mengxue Xu, Huan Zhou, Ning Sun, Li Zhang, Mengmeng Zhao, Jianhua He, Tingting Ran, Bo Sun, Weiwu Wang

**Affiliations:** a Department of Microbiology, College of Life Sciences, Nanjing Agricultural University, Nanjing 210095, People’s Republic of China; b Shanghai Synchrotron Radiation Facility, Shanghai Advanced Research Institute, Chinese Academy of Sciences, Shanghai 201204, People’s Republic of China

**Keywords:** PigF, induced fit, *O*-methyltransferases, conformational rearrangements, prodigiosin, crystal structure

## Abstract

Prodigiosin, a red linear tripyrrole pigment, is a typical secondary metabolite with numerous biological functions, such as anticancer, antibacterial and immunosuppressant activities, and is synthesized through a bifurcated biosynthesis pathway from 4-methoxy-2,2′-bipyrrole-5-carbaldehyde (MBC) and 2-methyl-3-*n*-amylpyrrole (MAP). The last step in the biosynthetic pathway of MBC is catalysed by PigF, which transfers a methyl group to 4-hydroxy-2,20-bipyrrole-5-carbaldehyde (HBC) to form the final product MBC. However, the catalytic mechanism of PigF is still elusive. In this study, crystal structures of apo PigF and *S*-adenosylhomocysteine (SAH)-bound PigF were determined. PigF forms a homodimer and each monomer consists of two domains: a C-terminal catalytic domain and an N-terminal dimerization domain. Apo PigF adopts an open conformation, while the structure of the complex with the product SAH adopts a closed conformation. The binding of SAH induces dramatic conformational changes of PigF, suggesting an induced-fit substrate-binding mechanism. Further structural comparison suggests that this induced-fit substrate-recognition mechanism may generally exist in *O*-methyltransferases. Docking and mutation studies identified three key residues (His98, His247 and Asp248) that are crucial for enzyme activity. The essential function of His247 and Asp248 and structure analysis suggests that both residues are involved in activation of the HBC substrate of PigF. The invariance of Asp248 in PigF further confirmed its essential role. The invariance and essential role of His98 in PigF suggests that it is involved in correctly positioning the substrate. This study provides new insight into the catalytic mechanism of PigF, reveals an induced-fit substrate-recognition model for PigF and broadens the understanding of *O*-methyltransferases.

## Abstract 2

Crystal structures of apo and SAH-bound PigF were determined and structural analyses revealed an induced-fit substrate-recognition model.

## 1. Introduction

*Serratia* spp. belong to the large family Enterobacteriaceae and are widely distributed in different environments such as soil, water and plant surfaces; some strains of *Serratia* are opportunistic pathogens of humans, plants and some insects (Hejazi & Falkiner, 1997 ▸). Prodigiosin is a typical secondary metabolite that is found in some *Serratia* strains (Lee *et al.*, 2011 ▸) and only appears in the late stages of bacterial growth. Many environmental factors have been demonstrated to influence the production of prodigiosin, including the availability of inorganic phosphate, the composition of the medium, the temperature and the pH (Williamson *et al.*, 2006 ▸). An anticancer function of prodigiosin has also been observed (Pérez-Tomás *et al.*, 2003 ▸).

**Figure 1:**
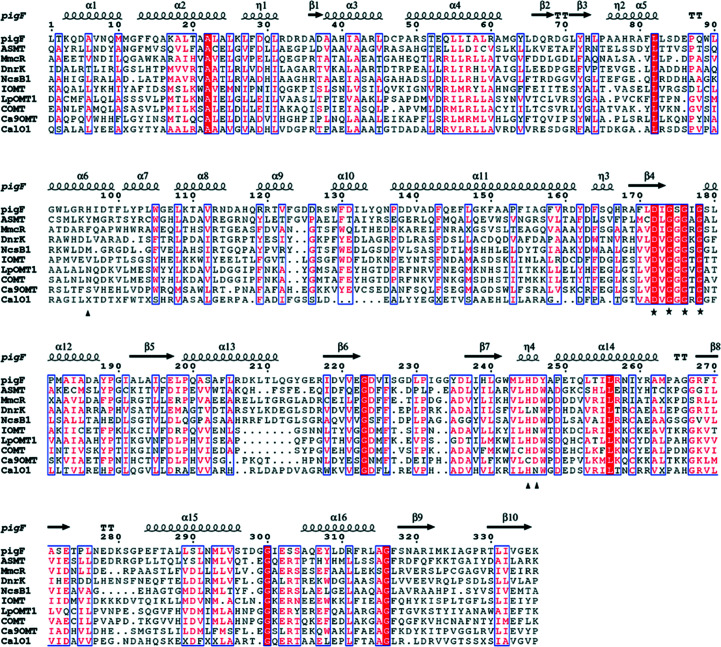
Sequence alignment of PigF with other methyltransferases. The conserved D*X*G*X*G*X*G motif is highlighted with a red background and is indicated by stars, conservatively substituted residues are boxed and His98, His247, Asp248 of PigF are indicated by triangles. The secondary structure of PigF is depicted above the alignment.

**Figure 2:**
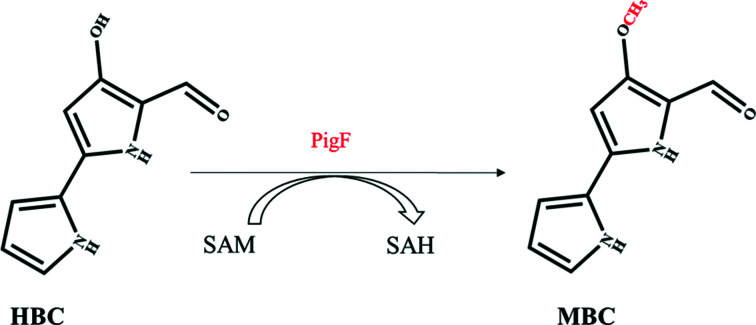
The last step in the MBC biosynthesis pathway: PigF transfers a methyl group to HBC to form the final product MBC.

The prodigiosin biosynthetic gene clusters from various *Serratia* strains have been cloned and sequenced (Harris *et al.*, 2004 ▸) and typically include 14 genes. These gene clusters were also functionally expressed in heterologous hosts such as *Escherichia coli* and *Erwinia carotovora* subsp. *carotovora* (Thomson *et al.*, 2000 ▸; Harris *et al.*, 2004 ▸). The biosynthesis of prodigiosin is proposed to take place via a bifurcated pathway in which 4-methoxy-2,2′-bipyrrole-5-carbaldehyde (MBC) and 2-methyl-3-*n*-amylpyrrole (MAP) are separately synthesized before being condensed to form prodigiosin (Morrison, 1966 ▸; Wasserman *et al.*, 1973 ▸; Harris *et al.*, 2004 ▸; Williamson *et al.*, 2005 ▸). PigB, PigD and PigE have been demonstrated to synthesize MAP, while the remaining gene products in the cluster, with the exception of PigC (which condenses MAP and MBC to synthesize prodigiosin), are involved in the biosynthesis of MBC. PigA, PigG, PigH, PigI and PigJ have been demonstrated *in vitro* to participate in the synthesis of 4-hydroxy-2,2′-bipyrrole-5-methanol (HBM; Garneau-Tsodikova *et al.*, 2006 ▸). HBM is then converted to 4-hydroxy-2,20-bipyrrole-5-carbaldehyde (HBC) by oxidization by PigM. PigF, which is a putative *O*-methyltransferase (Fig. 1 ▸), catalyses the final step in the biosynthetic pathway of MBC by transferring a methyl group to HBC to form the final product MBC (Williamson *et al.*, 2005 ▸, 2006 ▸; Fig. 2 ▸). Although a functional study showed that PigF participates in the methylation of HBC to form MBC, no structural information is available for this protein. To elucidate the mechanism of the final step of MBC biosynthesis, we cloned and expressed the *pigF* gene from *S. marcescens* FS14 in *E. coli* and crystallized the PigF protein (Liu *et al.*, 2012 ▸). Here, we present the crystal structures of PigF at 1.9 Å resolution and of PigF in complex with the product *S*-adenosylhomocysteine (SAH) at 1.97 Å resolution. Structural comparison of these two structures revealed an induced-fit mechanism for substrate recognition, while docking and mutational results identified three key residues in PigF.

## 2. Materials and methods

### 2.1. Bacterial strains, plasmids and culture conditions

The bacterial strains and plasmids used in this study are listed in Supplementary Table S1. *S. marcescens* FS14 and the mutant FS14ΔPigF were grown in Luria–Bertani (LB) medium (1% tryptone, 0.5% yeast extract and 1% NaCl) at 28°C. The complementary strains were grown in LB medium at 28°C supplemented with an appropriate antibiotic when necessary. *E. coli* strains were grown in LB medium at 37°C supplemented with kanamycin. Antibiotics were supplemented (30 µg ml^−1^ kanamycin for protein expression; 60 µg ml^−1^ kanamycin and 15 µg ml^−1^ tetracycline for mutant selection) when necessary.

### 2.2. Construction of in-frame deletion mutant

The homologous arms (upstream of *pigF* and downstream of *pigF*) were amplified by two-step overlapping PCR (Ho *et al.*, 1989 ▸) and were then cloned into the suicide plasmid pWDF (Wu *et al.*, 2016 ▸). The plasmid was introduced into wild-type FS14 by triparent conjugation. The in-frame deletion mutant of *pigF* was constructed by homologous recombination and was identified by PCR and DNA sequencing. The primers used in construction and verification are listed in Supplementary Table S2.

### 2.3. Point-mutant construction

The *pigF* single or double point-mutant genes were amplified by two-step overlapping PCR (Ho *et al.*, 1989 ▸) and cloned into pMTKQS (constructed by removing the NcoI site and the fragment between NcoI and NdeI in pMTK; Li *et al.*, 2018 ▸) to generate the overexpression plasmids. These plasmids were then transferred into FS14ΔPigF to perform the complementation assay. The primers used in construction are listed in Table S2.

### 2.4. Prodigiosin measurement

The mutant and complementary strains were cultured in 3 ml LB broth for 12 h at 28°C and 180 rev min^−1^ and were subcultured in 20 ml LB broth for 10 h at 28°C and 180 rev min^−1^. They were finally transferred to 50 ml glycerol peptone broth (1% glycerol, 1.5% peptone) and cultured at 28°C and 180 rev min^−1^, and supplemented with kanamycin (30 µg ml^−1^) when necessary. The cells from a 500 µl culture were harvested by centrifugation at 15 000 rev min^−1^ for 15 min. The cell pellets were resuspended in 5 ml acidified alcohol (4% 1 *M* HCl in alcohol) to extract prodigiosin at 28°C and the absorbance of the supernatant was measured at 220–700 nm (Slater *et al.*, 2003 ▸). All experiments in this work were performed independently three times.

### 2.5. Gene cloning, protein expression and purification

The *pigF* gene from *S. marcescens* FS14 was amplified from genomic DNA using colony PCR. The amplified DNA fragment was then cloned into the expression vector pET-24b (Novagen) to generate the expression construct as described previously (Liu *et al.*, 2012 ▸). The constructed plasmid was verified by DNA sequencing and then transformed into *E. coli* strain C43 (DE3) cells for protein expression. The freshly transformed colony was inoculated overnight in LB broth supplemented with 30 µg ml^−1^ kanamycin at 37°C and 180 rev min^−1^. The overnight cultures of the transformants were then diluted 1:50 and grown to 1.0 absorbance units at 600 nm at 37°C. The cells were induced with 0.5 m*M* isopropyl β-d-1-thiogalactopyranoside (IPTG) at 30°C for 3 h and were harvested by centrifugation at 5000 rev min^−1^ for 10 min. The target proteins were then purified as described previously (Liu *et al.*, 2012 ▸).

### 2.6. Crystallization, data collection and structure determination

Selenomethionine-derivatized (SeMet) PigF was crystallized by the hanging-drop vapour-diffusion method in space group *P*12_1_1 (Liu *et al.*, 2012 ▸). X-ray diffraction data were collected on the BL17U beamline at the Shanghai Synchrotron Radiation Facility (SSRF) at 100 K and were processed using *XDS* (Kabsch, 2010 ▸). The structure of PigF without the SAH cofactor was solved by the Se-SAD method. Experimental phasing was performed with *SHELXD*/*SHELXE* (Sheldrick, 2015 ▸) using *HKL*2*MAP *(Pape & Schneider, 2004 ▸). Electron-density modification was performed with *SHELXE* and the model was autobuilt with *ARP*/*wARP* (Langer *et al.*, 2008 ▸). Manual model adjustment was performed with *Coot* (Emsley *et al.*, 2010 ▸) and structure refinement was carried out using *REFMAC*5 (Murshudov *et al.*, 2011 ▸) and *Phenix* (Liebschner *et al.*, 2019 ▸). To obtain the structure of PigF complexed with SAH or *S*-adenosylmethionine (SAM), PigF was premixed with SAH or SAM for 30 min and then subjected to crystallization using the same conditions as used for apo PigF [pH 4.6 and 12% 2-methyl-2,4-pentanediol (MPD)]. Crystals suitable for diffraction were obtained for PigF crystallized with both SAH and SAM. The crystals belonged to space group *P*12_1_1 with unit-cell parameters *a* = 69.1, *b* = 52.36, *c* = 92.83 Å, β = 97.35° for native PigF and space group *P*12_1_1 with unit-cell parameters *a* = 43.9, *b* = 109.2, *c* = 63.9 Å, β = 93.1° for the PigF–SAH complex. The structure of PigF co-crystallized with SAH or SAM was solved by molecular replacement using the apo PigF structure as the template with *Phaser *(McCoy *et al.*, 2007 ▸). *PyMOL* was used to prepare figures depicting structures (DeLano, 2002 ▸). The data-collection, processing and structure-refinement statistics are summarized in Supplementary Table S3.

### 2.7. Docking

The docking of MBC into the structure of the PigF–SAH complex was performed by *AutoDock Vina* (Trott & Olson, 2010 ▸). PDBQT files for the protein and the product MBC were prepared using *MGL Tools* (http://mgltools.scripps.edu/downloads). Chain *A* of the PigF–SAH complex structure was used to generate the protein PDBQT file, while the PDB file for MBC was generated with *JLigand* in *Coot* and then used to prepare the PDBQT file. The grid box centre is *x* = 4.806, *y* = −16.306, *z* = 3.139, with dimensions *x* = 30, *y* = 26, *z* = 24 Å.

### 2.8. Data availability

The structures presented in this paper have been deposited in the Protein Data Bank (PDB) as entries 7clf and 7clu.

## 3. Results

### 3.1. PigF is a member of the AdoMet-MTase superfamily and is essential for prodigiosin biosynthesis

The PigF protein consists of 338 amino-acid residues with a predicted molecular mass of 37.6 kDa and its encoding gene is located in the *pig* (prodigiosin synthesis) gene cluster transcribed as a polycistronic mRNA. It shares 100% sequence identity with PigF from *S. marcescens* VGH107 (NCBI accession No. WP 004940222.1). Purified recombinant PigF showed a similar molecular weight to that predicted. A *BLAST* search result revealed that PigF has a putative *S*-adenosylmethionine binding site (D*X*G*X*G*X*G) and belongs to the *S*-adenosylmethionine-dependent methyltransferase (SAM or AdoMet-MTase) superfamily. The *BLAST* search also retrieved several homologs from *Serratia* species with sequence identities of 83–100% and corresponding SAM binding sites. We also found several homologs from other species, such as *Pseudoalteromonas rubra*, *Vibrio gazogenes*, *Hahella chejuensis* and *Zooshikella ganghwensis*, with sequence identities of 47–80%, that also contained the same SAM binding site. Sequence analysis showed the C-terminal domain of PigF (residues 165–338) to be the putative catalytic domain, which shares homology with the AdoMet-MTase family proteins, while the N-terminal domain (residues 1–164) is a dimerization domain.

**Figure 3:**
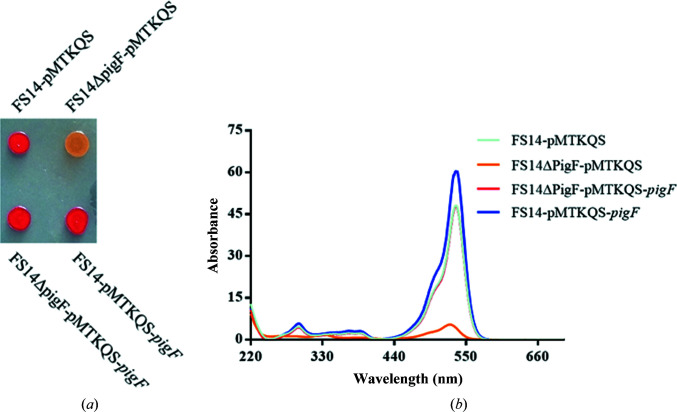
Absorption spectra of prodigiosin and norprodigiosin from FS14 and the FS14ΔPigF mutant. (*a*) The colour change of the products between FS14 and FS14ΔPigF. (*b*) The maximum absorption wavelength of prodigiosin from FS14-pMTKQS (cyan), the complementary strain FS14ΔPigF-pMTKQS-*pigF* (red) and the overexpression strain FS14-pMTKQS-*pigF* (blue) and of norprodigiosin from FS14ΔPigF-pMTKQS (orange).

To elucidate the function of PigF, an in-frame deletion mutant of the *pigF* gene was constructed. Measurement of the prodigiosin produced by the wild type and the PigF deletion mutant showed that the deletion of PigF dramatically affected the biosynthesis of prodigiosin. The colour of the mutant colonies became pale yellow compared with the red colour for wild-type FS14. The introduction of a plasmid carrying an intact *pigF* gene restored the biosynthesis of prodigiosin when PigF was induced with IPTG. This result indicated that PigF is essential for the biosynthesis of prodigiosin in FS14. Further analysis of the production of prodigiosin by mutant and wild-type FS14 showed that the PigF deletion mutant was still able to produce a prodigiosin-like product but that it had different properties to prodigiosin [Fig. 3 ▸(*a*)]. The mutant could only synthesize an orange product which had a maximum absorption wavelength at 525 nm, compared with the wild type which produces prodigiosin with a maximum absorption wavelength at 535 nm. The maximum absorption of the product produced by the PigF deletion mutant is blue-shifted by about 10 nm compared with prodigiosin [Fig. 3 ▸(*b*)]. The orange product was proposed to be nonmethylated prodigiosin, and this is consistent with previous results observed using *Serratia* sp. 39006 (Wilf & Salmond, 2012 ▸; Williamson *et al.*, 2005 ▸). Besides the wavelength difference, the amount of product produced by the PigF deletion mutant is only around 12.6% of that of wild-type FS14 [Fig. 3 ▸(*b*)]. This result also suggests that even without the methylation of HBC PigC can still condense HBC and MAP to form a prodigiosin-like product, but the relative activity is much lower than that for the condensation of MBC and MAP.

### 3.2. Structure determination and overall structure of apo PigF

**Figure 4:**
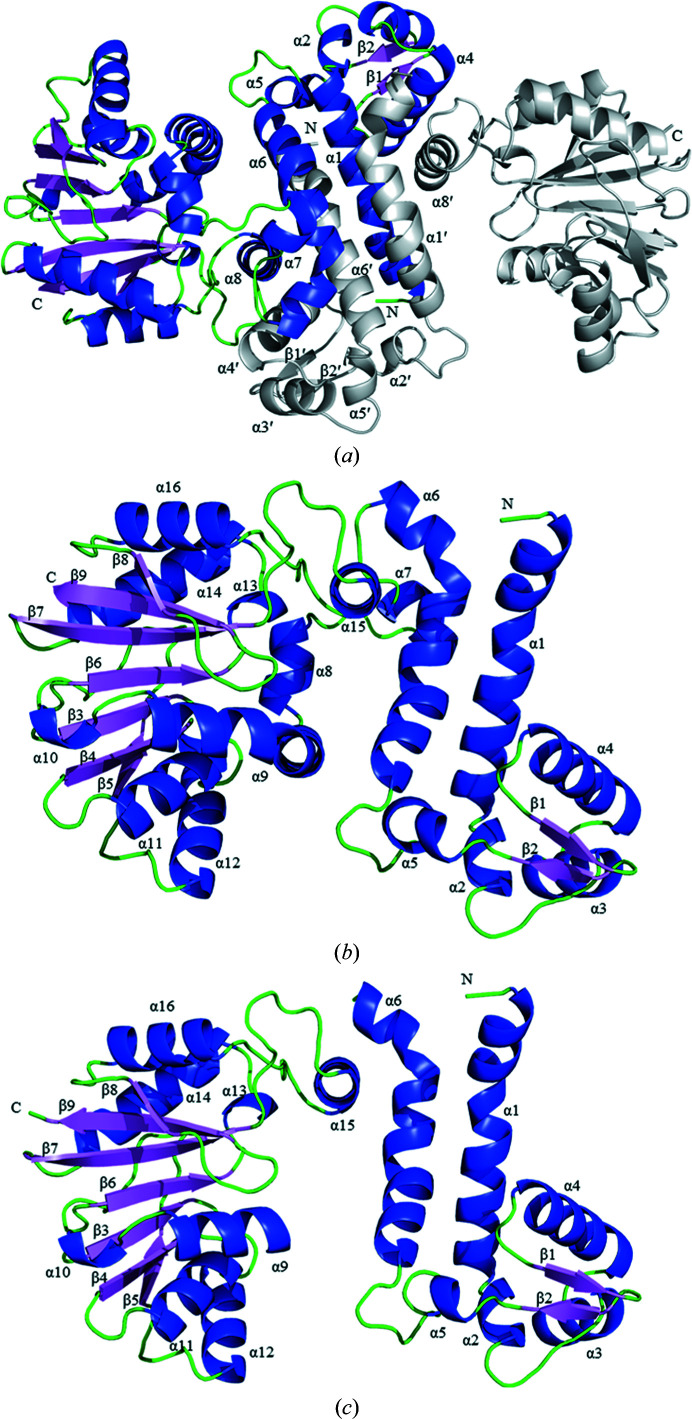
Overall structure of apo PigF. (*a*) The structure of the PigF homodimer. The helices, sheets and loops of monomer *A* are shown in blue, violet and green, respectively; monomer *B* is coloured grey. Secondary-structure elements involved in the dimer interface are labelled. (*b*) Chain *A* of PigF; helices, sheets and loops are shown in blue, violet and green, respectively. (*c*) Chain *B* of PigF, which lacks residues 117–152; helices are coloured blue, strands are coloured violet and loops are shown in green.

Recombinant PigF was expressed in *E. coli* as an N-terminally polyhistidine-tagged protein and was purified by Ni^2+^-affinity chromatography and molecular-exclusion chromatography. After purification, the protein was crystallized by vapour diffusion in hanging drops using MPD as the precipitant. Crystal structure determination of PigF was first attempted by molecular replacement (MR) but was not successful. We therefore prepared SeMet PigF crystals and collected a complete 1.9 Å resolution data set to solve the crystal structure. The apo PigF crystal belonged to space group *P*12_1_1 (unit-cell parameters *a* = 69.1, *b* = 52.36, *c* = 92.83 Å, β = 97.35°) and contained two molecules in the asymmetric unit that formed a stable dimer. It is notable that the two chains in the final model are somewhat different: no electron density was observed in the region of residues 117–152 for chain *B*, corresponding to α7, α8 and a small part of α9 in addition to two loop regions [Fig. 4 ▸(*c*)], while the other parts of the structure are almost identical, with a root-mean-square deviation (r.m.s.d.) of 0.88 Å when superimposed. PigF contains two domains: an N-terminal dimerization domain and a C-terminal catalytic domain. The C-terminal domain comprising amino-acid residues 118–338 consists of a set of alternating β-strands and α-helices which form a Rossmann-like fold. This Rossmann-like fold is built up of a central mainly parallel β-sheet packed against eight alternating α-helices (α8–α14 and α16), while the β-sheet is composed of seven β-strands (β3–β9) which are parallel to each other, with the exception of β9 which is antiparallel [Fig. 4 ▸(*b*)]. The N-terminal dimerization domain consists of residues 3–117. This domain is mainly composed of helices (α1–α6) with two short antiparallel β-strands (β1–β2) located between α4 and α5. The core of the dimerization domain of PigF consists of a bundle of four (2 × 2) long α-helices from two monomers, which are bent and tightly intertwined. This bundle is surrounded by several other shorter helices plus two short β-strands located at the extremities. The secondary elements that are mainly engaged in dimer formation include helices α1, α2, α3, α4, α5, α6 and α15 and β-strands β1 and β2 [Fig. 4 ▸(*a*)]. The interaction area of the dimer interface is very extensive and covers 3466 Å^2^, mostly being dominated by hydrophobic interactions together with 23 hydrogen bonds and seven salt bridges, thus resulting in a very stable dimer. This is consistent with the result in solution as determined by comparison with standards in a nondenaturing gel (Supplementary Fig. S1).

### 3.3. The structure of the complex of PigF with the cofactor SAH

**Figure 5:**
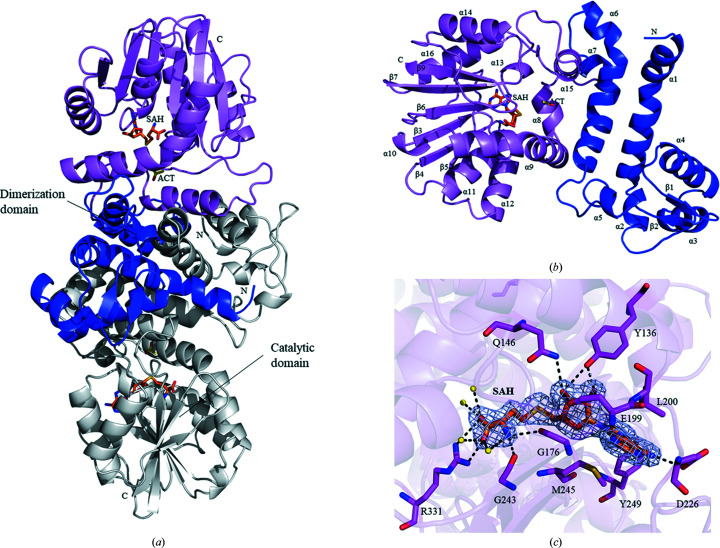
The structure of the complex of PigF with the cofactor SAH. (*a*) The structure of the PigF dimer present in the asymmetric unit with the cofactor SAH. The dimerization domain of subunit *A* is coloured blue and the catalytic domain is coloured violet. Subunit *B* is coloured grey. SAH and acetate (ACT) are shown as orange and yellow sticks, respectively. (*b*) Chain *A* of PigF with the cofactor SAH. Individual domains are coloured blue and violet, respectively. SAH (C atoms in orange) is shown in the SAH binding domain. (*c*) The SAH binding site. SAH is coloured orange, residues are shown as magenta sticks, water molecules are shown as yellow spheres and hydrogen bonds are shown as dotted lines and coloured black; the cartoon representation of the PigF–SAH structure is shown with 80% transparency.

To reveal how the cofactors SAH and SAM bind to PigF, SAH or SAM was mixed with the protein at a final concentration of 2 m*M* before crystallization. In a similar way, the proteins were crystallized by vapour diffusion in hanging drops using MPD as the precipitant. Similar to apo PigF, crystals appeared from PigF mixed with SAM just a few hours after crystallization setup. These crystals belonged to the same space group, *P*12_1_1, with similar unit-cell parameters as apo PigF. Structure determination showed that the structure is same as that of apo PigF, with no electron density observed for the SAM cofactor. For the PigF–SAH complex, long narrow thin plate-shaped crystals appeared several days later. Surprisingly, the shape of the crystals was completely different from those of apo PigF. The crystals also belonged to space group *P*12_1_1, but with very different unit-cell parameters, *a* = 43.9, *b* = 109.2, *c* = 63.9 Å, β = 93.1°, compared with the apo PigF crystals (*a* = 69.1, *b* = 52,36, *c* = 92.83 Å, β = 97.35°), indicating that the apo PigF and PigF–SAH crystals are differently formed. The crystal structure was determined by the MR method using apo PigF as the template, and the resulting phased map revealed clear electron density for the entire protein molecule and the SAH cofactor due to its relative high resolution; subsequent refinement yielded an *R*_work_ of 18.53% and an *R*_free_ of 25.34%. The asymmetric unit contained two monomers of PigF [Fig. 5 ▸(*a*)], which form a stable dimer similar to that of apo PigF. In constrast to apo PigF, both chains consist of residues 3–338, with the region that is missing in one monomer of apo PigF being clearly resolved in PigF–SAH; the two subunits are almost structurally identical, with a backbone r.m.s.d. of 0.70 Å. The only remarkable difference between chain *A* and chain *B* is that the antiparallel β-strands (β1 and β2) in chain *A* were displaced by loops in chain *B*. Similar to apo PigF, each monomer is composed of two domains: a C-terminal catalytic domain (residues 118–338) and an N-terminal dimerization domain (residues 3–117). The cofactor SAH is located in the C-terminal catalytic domain [Figs. 5 ▸(*a*) and 5 ▸(*b*)].

### 3.4. SAH binding site

The C-terminal domain of PigF contains the conserved D*X*G*X*G*X*G fingerprint needed for the binding of the SAM or SAH cofactor (Fig. 1 ▸). In the PigF–SAH complex structure determined here, the cofactor product SAH is well defined by electron density. Fig. 5 ▸(*c*) clearly shows the binding of an SAH molecule in the cofactor site. The cofactor binding site is mainly built up by residues from the C-terminal catalytic domain. Based on this structure, SAH is bound to PigF in a similar manner as in other small-molecule methyltransferases. The cofactor interacts with the enzyme via an extensive hydrogen-bonding network and a few hydrophobic inter­actions. The adenine ring is packed against Met245 and Leu200 from both sides, and the N6 and N7 amino groups form hydrogen bonds to Asp226 and Tyr249, respectively [Fig. 5 ▸(*c*)]. The ribose moiety is anchored to PigF through hydrogen bonds from the O2′* and O3′* hydroxyl groups to the side chains of Tyr136, Gln146 and Glu199. The terminal N1 group forms strong hydrogen bonds to the backbone carbonyl groups of Gly176 (the first glycine in the conserved D*X*G*X*G*X*G motif) and Gly243, while the terminal carboxyl group forms a salt bridge with Arg331 [Fig. 5 ▸(*c*)].

### 3.5. Structural comparison between the apo and SAH-bound PigF structures reveals an induced-fit mechanism

To analyse the structural changes on the binding of SAH, a structural superposition of apo PigF and PigF–SAH was performed. Since chain *B* of apo PigF contains a region that is not resolved in the crystal structure, we superimposed chain *A* with the structure of PigF–SAH. The superposition yields a backbone r.m.s.d. of 1.48 Å, suggesting that some structural changes take place when SAH binds to PigF. Since PigF has two domains, the N-terminal dimerization domain and the C-terminal catalytic domain [Fig. 5 ▸(*a*)], we further superposed both domains separately. Superposition of the N-terminal domain yields an r.m.s.d. of only 0.44 Å, indicating that this domain is almost identical in the two structures, whereas superposition of the C-terminal catalytic domain results in an r.m.s.d. of 1.77 Å, indicating that large conformational changes occur in the catalytic domain on the binding of SAH.

**Figure 6:**
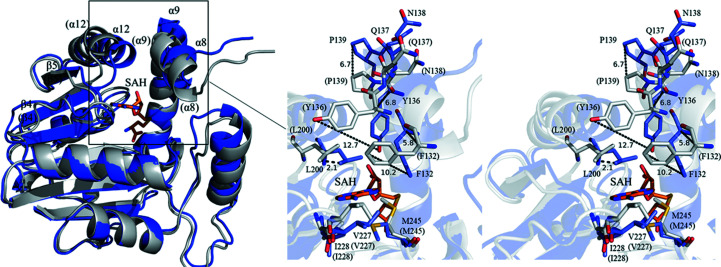
Conformational changes: structure comparison of PigF–SAH and apo PigF. The cartoon and ribbon representation of PigF–SAH is coloured blue, residues of PigF–SAH are coloured blue and labelled in black. The cartoon and ribbon representation of apo PigF is coloured grey, residues are coloured grey and labelled in black in parentheses. SAH is shown as orange sticks.

The residues constituting the SAH binding pocket undergo dramatic conformational rearrangements on the binding of the ligand (Fig. 6 ▸). The side chain of Met245 swings about 100° to pack against the purine ring of SAH from one side, while at the same time the loop region harbouring Leu200 moves towards SAH by around 2.5 Å and the side chain of Leu200 packs against the purine ring from the other side (Fig. 6 ▸). Interestingly, helix α8 undergoes a 90° counterclockwise rotation and a forward movement of about 2.5–4.0 Å on the binding of SAH. The largest movement of the backbone in helix α8 is of Tyr136, which moves around 6.8 Å (C^α^); the side chain of Tyr136 tilts and the hydroxyl group hydrogen bonds to O2′ of the ribose group with a movement of about 12 Å of the hydroxyl group. Another large movement is made by Phe132, which packs against the ribose ring by moving around 5.8 Å (C^α^). The side chain of Phe132, along with those of Trp249, Ile228 and Val227, packs perpendicularly against the adenosine ring of SAH. The linker (residues 137–139) connecting helices α8 and α9 moves away from SAH to avoid clashing with helix α8 (Fig. 6 ▸). The region from the C-terminus of β4 to the N-terminus of helix α12 moves towards SAH to generate interactions with SAH, including hydrogen bonds between Glu199 and O2′ and O3′ of the ribose ring, with Leu200 packing against the adenosine ring. The SAH is tightly bound to the enzyme and is not accessible to solvent after the conformational changes that take place on product binding. The structural comparison of apo PigF and PigF–SAH indicates that the active site of apo PigF is nonproductive. The observed structural differences in the two structures indicated that such conformational changes could involve domain rotation and loop closure, leading to open and closed active-site conformations.

### 3.6. Structural comparison of PigF with other methyltransferases

**Figure 7:**
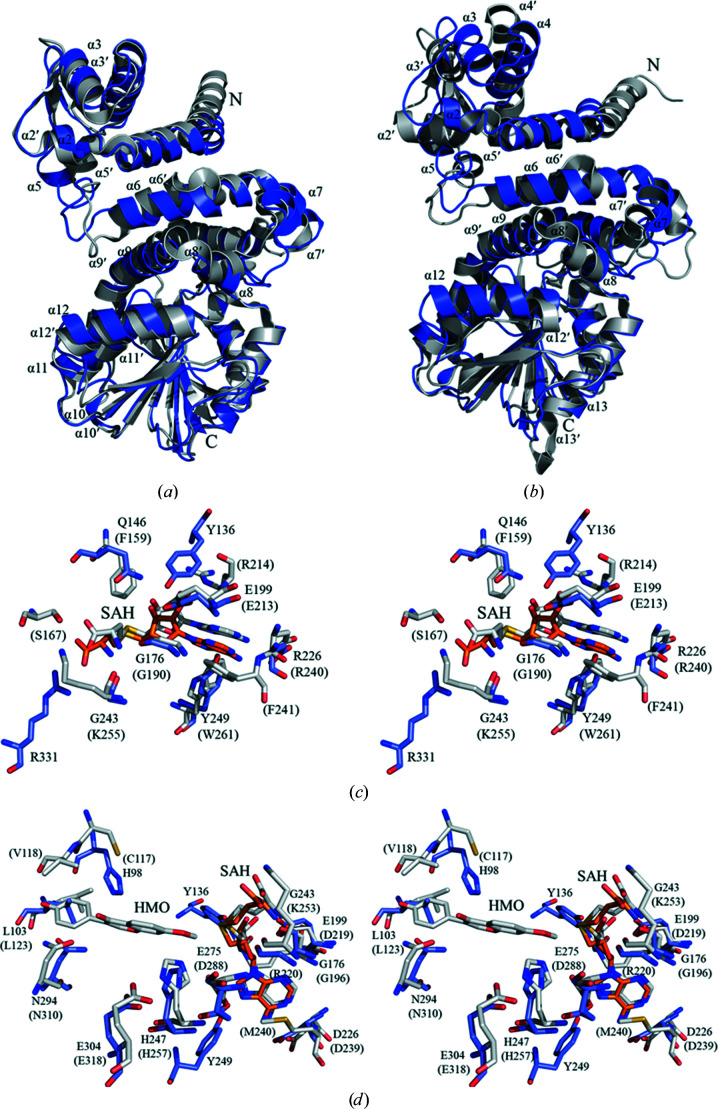
Structural comparison of the active site of PigF with those of MmcR and IOMT. (*a*, *b*) Structure comparison of PigF–SAH with MmcR (PDB entry 3gwz) (*a*) and IOMT (PDB entry 1fp2) (*b*). PigF–SAH is coloured blue and MmcR and IOMT are coloured grey. (*c*, *d*) Diagrams showing the superimposed binding sites of PigF–SAH and MmcR (*c*) and IOMT (*d*). The residues and SAH in PigF–SAH are coloured blue and orange, respectively, and labelled in black; the residues and SAH in MmcR and IOMT are coloured grey and labelled black in parentheses.

The structures with the highest *Z*-scores retrieved by a *DALI* search were *N*-acetyl­serotonin methyltransferase (ASMT; PDB entry 4a6d; Botros *et al.*, 2013 ▸), mitomycin-7-*O*-methyltransferase (MmcR; PDB entry 3gwz; Singh *et al.*, 2011 ▸), carminomycin 4-*O*-methyltransferase (DnrK; PDB entry 1tw3; Jansson *et al.*, 2004 ▸), neocarzinostatin *O*-methyltransferase (NcsB1; PDB entry 3i58; Cooke *et al.*, 2009 ▸) and aclacinomycin 10-hydroxylase (RdmB; PDB entry 1xds; Jansson *et al.*, 2005 ▸), with *Z*-scores of 32.9, 30.8, 30.6, 30.0 and 28.6, respectively (and with r.m.s.d.s of 2.7, 2.5, 2.7, 2.9 and 3.0 Å, respectively). Despite the low sequence identities (<27%) between PigF and these homologues, PigF shares the same fold with them. PigF has the highest sequence identity of 27% to ASMT, with an r.m.s.d. of 2.7 Å when superposed, and has a sequence identity of 23% to MmcR, with an r.m.s.d. of 2.5 Å (Supplementary Table S4). There are also some significant differences in the structural comparison with other methyltransferases. The most straightforward difference is the α10 helix, which is only present in MmcR [Fig. 7 ▸(*a*)]. In the overall SAH binding pockets of PigF and MmcR, we found differences in the cofactor pocket. The most obvious is the loop that interacts with the homocysteine and ribosyl moiety of SAH situated between α8 and α11 (which contains the glycine-rich consensus sequence D*X*G*X*G*X*G) and an acidic residue in the loop between β5 and β6 (Asp226 in PigF), which interacts with the exocyclic N6 of the adenine ring of the cofactor. Tyr249 forms a hydrogen bond to N7 of the adenine ring which does not exist in MmcR [Fig. 7 ▸(*c*)]; in MmcR this position is occupied by the side chain of Phe241.

Compared with isoflavone *O*-methyltransferase (IOMT; PDB entry 1fp2; Zubieta *et al.*, 2001 ▸), we found that the dimer domain α2–α5 of PigF is more compact than that of IOMT, α12 of PigF is longer than that of IOMT, and α10 and α13 of PigF are shorter than those of IOMT [Fig. 7 ▸(*b*)]. We also found that the SAH binding pocket contains the conserved sequence D*X*G*X*G*X*G and an acidic residue (Asp226 in PigF) that interacts with the exocyclic N6 atom of the adenine ring of the cofactor. In IOMT, the substrate-binding pocket contains the conserved residues His257, Asn310 and Glu318 (corresponding to His247, Asn294 and Glu304 in PigF) [Fig. 7 ▸(*d*)].

### 3.7. Docking and site-directed mutation

**Figure 8:**
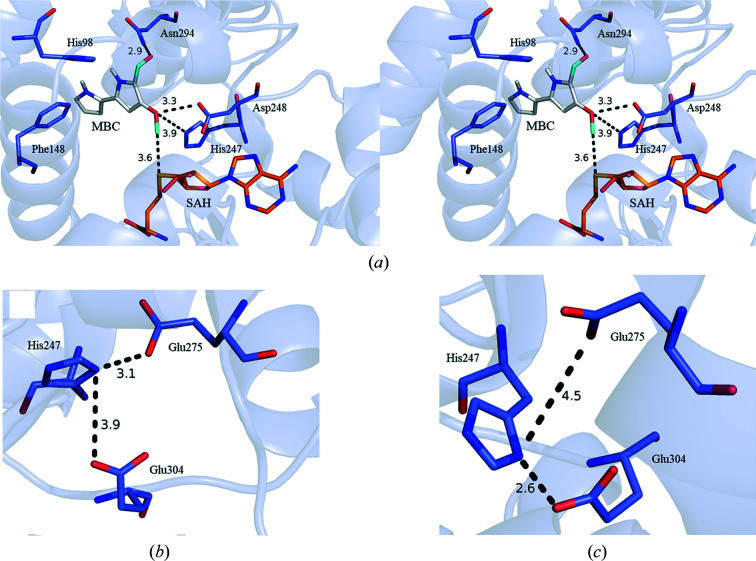
Docking model of PigF–SAH–MBC. (*a*) The putative substrate-binding pocket for MBC in the docking model of PigF–SAH–MBC. (*b*) The conformation of His247 in monomer *A*. (*c*) The conformation of His247 in monomer *B*. The PigF–SAH–MBC structure is shown as a blue cartoon. MBC, SAH and the residues and hydrogen bonds in PigF–SAH–MBC are coloured grey, orange, blue and black, respectively. The cartoon representation of the PigF–SAH–MBC structure is shown at 80% transparency.

To elucidate the catalytic mechanism of PigF, we performed a docking experiment of MBC into the PigF–SAH complex. The best model, with an affinity of −6.2 kcal mol^−1^, was used for analysis. In this docking model, MBC fits well into the putative substrate-binding pocket [Fig. 8 ▸(*a*)]. MBC was trapped in a relatively hydrophobic binding pocket constituted of Trp244, Phe152, Phe287, Phe148, Met13, Leu95, Phe16, Val9 and Leu291, with three hydrophilic residues (Ser298, Asn294 and His98) forming a patch in the binding pocket. The aldehyde group of MBC was located near Ser298 and Asn294, and His98 was located near the N atom of the first pyrrole ring, suggesting that they may be involved in positioning the substrate. His247 and Asp248 are located near the methyl group of MBC. To define the function of the putative catalytic residues (His247, Glu275 and Glu304), we constructed point mutants of these residues. An assay of the prodigiosin produced by these mutants indicated that His247 is essential for enzyme activity. The mutation of His247 to alanine abolished the enzyme activity, resulting in no production of prodigiosin (the absorption wavelength is very close to the absorption curve of the PigF deletion mutant) [Supplementary Fig. S2(*f*)]. Interestingly, mutation of Glu275 and Glu304, two conserved residues which bracket the catalytic histidine residue and were proposed to constrain the catalytic histidine residue in the proper position in chalcone *O*-methyltransferase (ChOMT; Zubieta *et al.*, 2001 ▸), did not have an obvious effect on the enzyme activity of PigF [Supplementary Figs. S2(*h*) and 2(*j*)], and the production of prodigiosin was similar to that of the wild-type strain. This result implied that these two glutamates are conserved in PigF but may not be involved in the activation of His247 or may not be as important as proposed in other methyltransferases such as ChOMT (Zubieta *et al.*, 2001 ▸). This result implied that PigF may use a different catalytic mechanism, although it has similar conserved residues in the catalytic centre as ChOMT. We also mutated Asp248, the residue immediately downstream of His247 located within the range of interaction with MBC. Similar to His247, mutation of Asp248 abolished the methyltransferase activity of PigF, no prodigiosin was synthesized and only norprodigiosin was observed [Supplementary Fig. S2(*g*)]. A double mutant exhibited the same phenomenon as the single mutants. This result indicated that Asp248 is essential for PigF function, as is His247. We also mutated other residues which are located near the docked MBC, including His98, Trp131, Asn294 and Ser298. The mutation of Trp131, Asn294 and Ser298 had no obvious effect on the function of PigF and the biosynthesis of prodigiosin was not affected by mutation of these residues [Supplementary Figs. S2(*i*), S2(*m*) and S2(*n*)]. Interestingly, mutation of His98, which does not make a direct interaction with MBC, abolished the function of PigF and only resulted in norprodigiosin [Supplementary Fig. S2(*e*)].

## 4. Discussion

### 4.1. PigF is a substrate induced-fit methyltransferase and this induced-fit mode may be common in *O*-methyltransferases

Prodigiosin is synthesized in a bifurcated pathway by accumulating MBC and MAP, which are then condensed by PigC. PigF has been demonstrated to catalyze the last step of the MBC pathway by transferring a methyl group to the hydroxyl group of HBC. We also demonstrated that deletion of PigF results in the formation of an orange variant of prodigiosin (Fig. 3 ▸), which is consistent with the result observed in *Serratia* 39006 (Wilf & Salmond, 2012 ▸), indicating the formation of norprodigiosin. Although an orange variant of prodigiosin could be synthesized by FS14ΔPigF, the amount synthesized was much lower than that synthesized by wild-type FS14 [Fig. 3 ▸(*b*)], indicating that PigC could recognize HBC but with a much lower efficiency compared with MBC. Thus, PigF is very important for the biosynthesis of prodigiosin, taking the amount of the final product prodigiosin into consideration. To reveal how PigF catalyzes the methyl transfer, here we determined two structures of PigF: those of apo PigF and SAH-bound PigF. Structure analysis and structural comparison with the structures of other methyltransferases indicate that PigF belongs to the typical *O*-methyltransferases (Fig. 4 ▸). However, structural comparison of apo PigF and PigF–SAH revealed that the binding of SAH, which is one of the products of PigF, induces dramatic conformational rearrangements (Fig. 6 ▸). These structural rearrangements took place at the catalytic site of the C-terminal domain and not in the N-terminal dimerization domain, indicating that the rearrangement is induced by the binding of SAH. The structural change induced by SAH results in the formation of a tight binding pocket for SAH and a putative substrate-binding pocket for HBC at the same time, suggesting that the two substrates (SAM and HBC) of the enzyme must be present at the same time to ensure that the reaction takes place, as only one substrate would induce the structural rearrangement. A similar large conformational change was observed in norcoclaurine-6-*O*-methyltransferase by Robin and coworkers; they also observed large conformational changes between the apo­enzyme and the SAH-bound enzyme and the structures did not undergo further conformational changes on the further addition of substrate or inhibitor (Robin *et al.*, 2016 ▸). The observation of this conformational rearrangement induced by substrate in different *O*-methyltransferases suggests that structural rearrangement may be a common feature of the *O*-methyltransferase family. However, further apo structures of different *O*-methyltransferases need to be characterized and compared with the holoenzyme structures to support this hypothesis; most of the presently solved structures of *O*-methyltransferases are not apo structures.

### 4.2. Catalytic mechanism and site-directed mutagenesis of residues in the active site

Two different mechanisms have been proposed for *O*-methyltransferases: acid/base catalysis for ChOMT and IOMT (Zubieta *et al.*, 2001 ▸) and the use of proximity as a catalytic tool in enzymes that lack a catalytic base (Jansson *et al.*, 2004 ▸). Structure-based sequence alignment showed that the putative catalytic residues (His247, Glu275 and Glu304, corresponding to His257, Asp288 and Glu318 in IOMT) are conserved in PigF, suggesting that PigF may use a similar acid/base catalytic mechanism to IOMT. We observed a dramatic structural change between apo PigF and PigF–SAH, and could observe a putative substrate-binding pocket near SAH, but we could not obtain ternary complexes with substrate or product due to the commercial unavailability of HBC. To gain further useful information to understand the mechanism of PigF, we performed a docking experiment using *AutoDock Vina* (Trott & Olson, 2010 ▸). Mutation of the putative catalytic residue His247 abolished the catalytic activity resulting in prodigiosin production (the absorption wavelength is very close to the absorption curve of the PigF deletion mutant) [Supplementary Fig. S2(*f*)], indicating that His247 is essential for enzyme activity. In contrast, mutation of Glu275 and Glu304 did not have an obvious effect on the enzyme activity of PigF [Supplementary Figs. S2(*h*) and S2(*j*)] and the production of prodigiosin was similar to that of the wild type. This result indicates that these two glutamates are conserved in PigF but may not be involved in the activation of His247 or may not be as important as proposed in other methyltransferases such as IOMT. In IOMT, His257 was bracketed by Asp288 and Glu318 and was constrained in the proper position by hydrogen bonding to Glu318 (Zubieta *et al.*, 2001 ▸). In our PigF structure His247 adopts two different conformations that are perpendicular to each other in monomer *A* and monomer *B*. The conformation of His247 in monomer *A* differs from that of His257 in IOMT. This different conformation of His247 prevents Glu304 from interacting with His247; in contrast, Glu275 forms a weak hydrogen bond to His247 [Fig. 8 ▸(*b*)]. In monomer *B* the conformation of His247 is identical to that of His257 in IOMT, and it also forms a hydrogen bond to Glu304 rather than Glu275 [Fig. 8 ▸(*c*)]. This subtle difference between the two monomers may imply that the active site may still need to undergo some subtle adjustments when binding the substrate.

In addition, the docking result showed that Asp248, the residue immediately downstream of His247, could also interact with the hydroxyl group of MBC [Fig. 8 ▸(*a*)]. Similar to His247, mutation of Asp248 abolished the methyltransferase activity of PigF [Supplementary Fig. S2(*g*)], and a His247 and Asp248 double mutant showed the same result as the single mutants [Supplementary Fig. S2(*k*)]. These results indicated that both His247 and Asp248 are essential for the function of PigF. We also compared PigF from different *Serratia* strains and other microorganisms, and found that Asp248 is conserved in PigF, emphasizing its importance. Based on the structural and mutational studies, we proposed that both His247 and Asp248 are involved in the deprotonation of HBC to generate a nucleophilic anion that attacks the methyl group of SAM in PigF to form the product MBC. Interestingly, the residues corresponding to Asp248 in other *O*-methyltransferases are also Asp or Asn, indicating that this residue is highly conserved. This highly conserved residue implies a crucial function of this residue in the enzyme activity of these enzymes (Fig. 1 ▸), as observed in PigF. Interestingly, mutation of His98, which does not make a direct interaction with MBC, abolished the function of PigF and resulted in only norprodigiosin [Supplementary Fig. S2(*e*)]. Sequence alignment of PigF enzymes showed that His98 is conserved (Supplementary Fig. S3). Taking these results together, we proposed that His98 may be involved in correctly positioning the substrate HBC and may be specific to PigF, as this residue is not conserved in other methyltransferases (Fig. 1 ▸).

In summary, our structural characterization of PigF provides a high-resolution structural framework for the substrate-binding mode of PigF. The dramatic conformational changes induced by binding of SAH revealed that PigF is a substrate induced-fit enzyme, and structural comparison suggested that this induced-fit substrate-recognition mechanism may be general to *O*-methyltransferases. The docking result and structure-based sequence alignment supports a catalytic base mechanism of PigF. Further mutational results confirmed the catalytic residue (His247) in PigF and also identified a highly conserved residue (Asp248) that is involved in the catalytic process.

Due to the unavailability of the substrate, we could not determine the enzyme kinetics of PigF and its mutants, and thus the activity of PigF and its mutants was determined from the production of prodigiosin. We still do not understand why the mutation of some residues in the binding pocket such as Asn294, Phe148, Ser298 and Trp131 does not have an effect on the biosynthesis of prodigiosin (Supplementary Fig. S2). These residues are not only conserved in PigF (Supplementary Fig. S3) but are also located in the substrate-binding pocket. Resolution of this question will depend on determining the structure of PigF in complex with substrate; this may be attempted by the crystallization of nonfunctional PigF protein (H247A, D248A) from a PigF deletion mutant because this protein may trap the substrate HBC.

## 5. Related literature

The following references are cited in the supporting information for this article: Chang *et al.* (2011 ▸), Li *et al.* (2015 ▸), Louie *et al.* (2011 ▸), Parsons *et al.* (2007 ▸), Wolters *et al.* (2013 ▸) and Zubieta *et al.* (2002 ▸).

## Supplementary Material

PDB reference: apo PigF, 7clu

PDB reference: SAH-bound PigF, 7clf

Supplementary Figures and Tables. DOI: 10.1107/S2052252521011696/jt5062sup1.pdf
