# Supplementary material for: Crystal structures of PigF, an O-methyltransferase involved in the prodigiosin synthetic pathway, reveal an induced-fit substrate-recognition mechanism
Source: IUCrJ. 2022 Mar 1;9(Pt 2):316–27. doi: 10.1107/S2052252521011696 (PMC8895007; doi:10.1107/S2052252521011696)
Supplement: Supplementary file 1 [file m-09-00316-sup1.pdf]

IUCrJ

Volume 9 (2022)

Supporting information for article:

Crystal structures of PigF, an *O*-methyltransferase involved in the prodigiosin synthetic pathway, reveal an induced-fit substrate-recognition mechanism

Shenshen Qiu, Dongqing Xu, Mengxue Xu, Huan Zhou, Ning Sun, Li Zhang, Mengmeng Zhao, Jianhua He, Tingting Ran, Bo Sun and Weiwu Wang

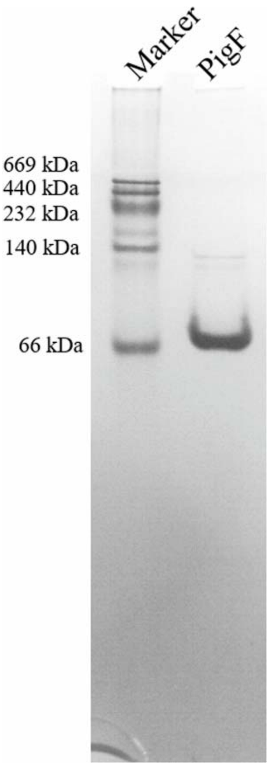

Figure S1 Determination of oligomeric state of PigF by non-denaturing gel.

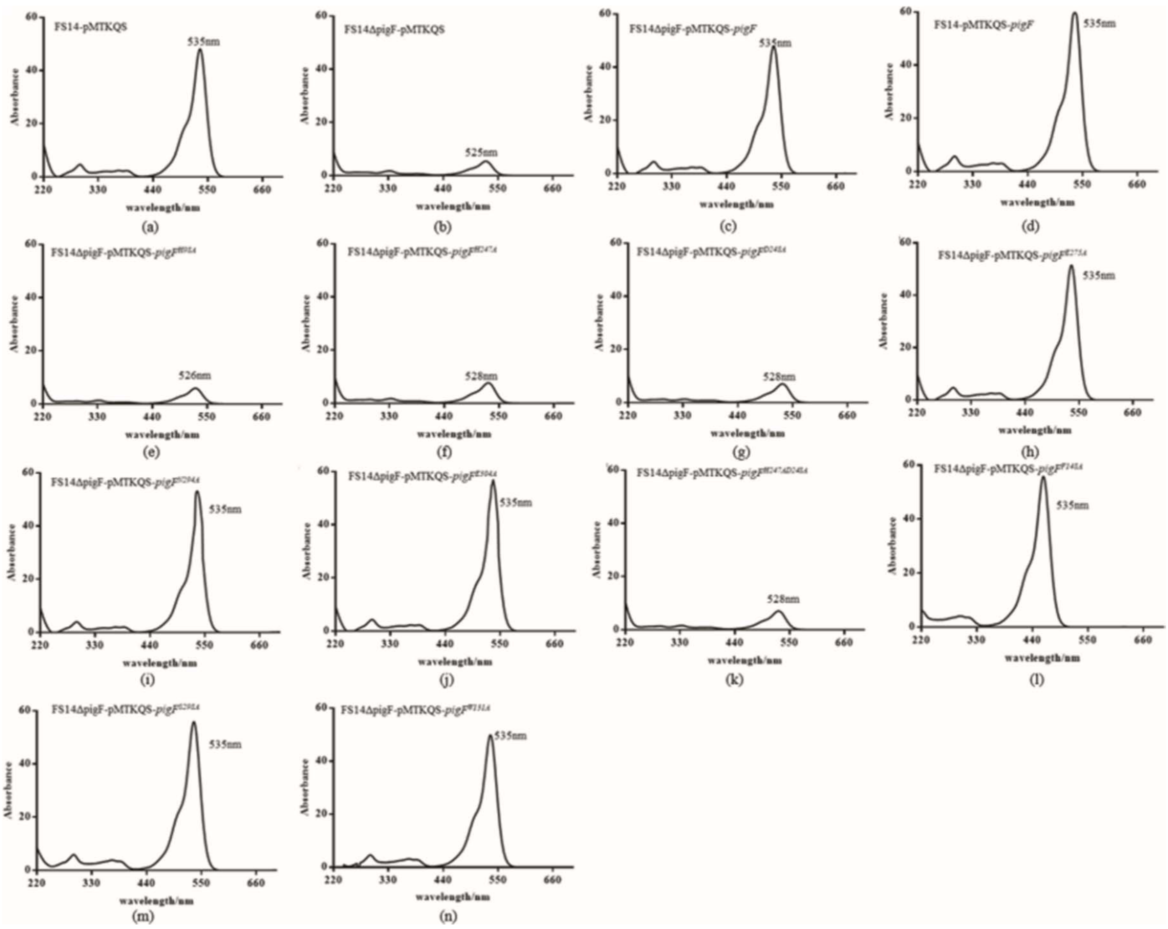

Figure S2 The maximum absorption wavelength of the prodigiosin from the different complementary strains. A-D represents the FS14-pMTKQS, the FS14ΔpigF-pMTKQS, the complementary strains FS14ΔpigF-pMTKQS-*pigF* and the overexpress strain FS14-pMTKQS-*pigF*; E-N represents the H98A, H247A, D248A, E275A, N294A, E304A, H247AD248A, F148A, S298A and W131A point mutant complementary strains, respectively.

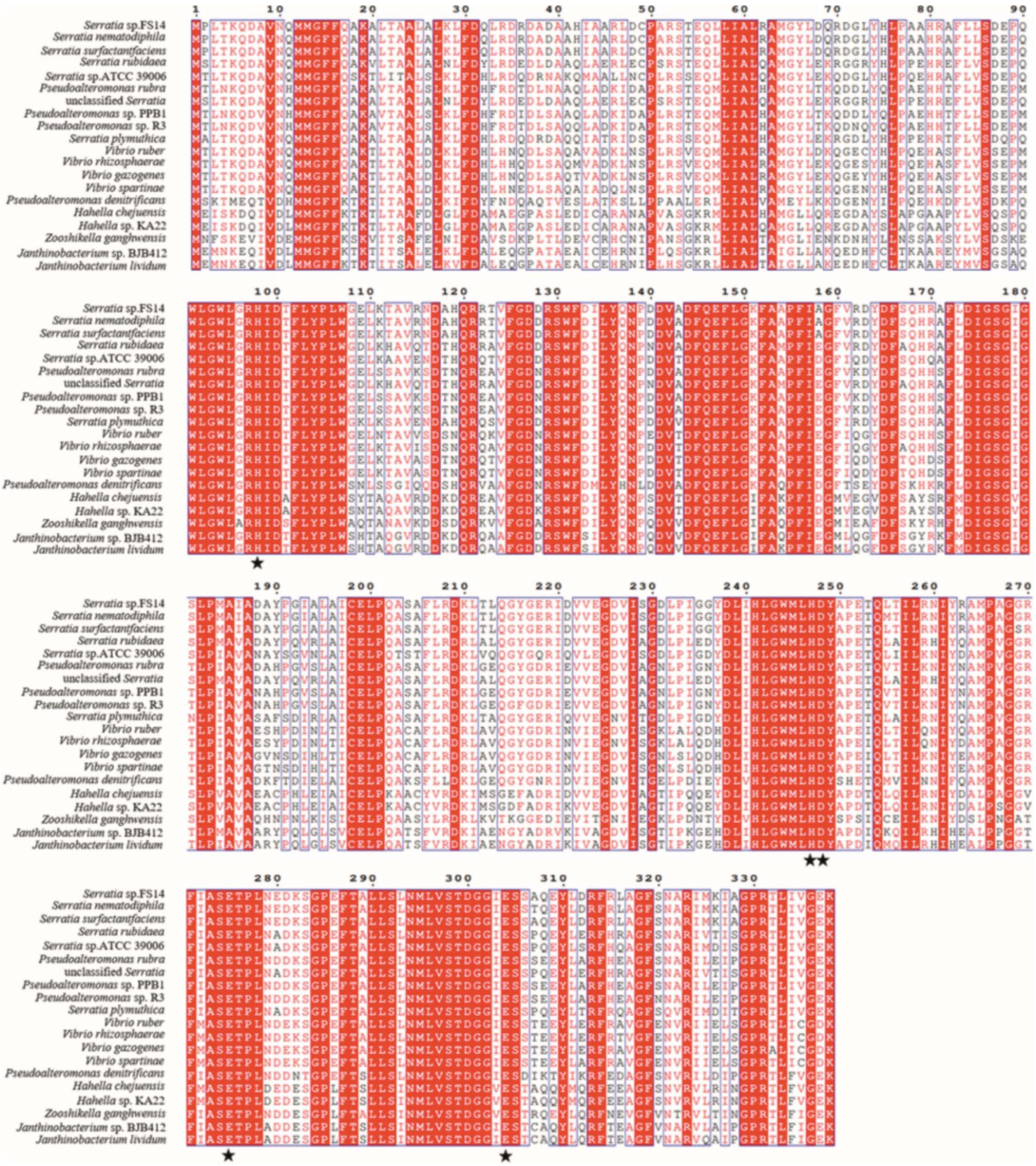

Figure S3 Sequence alignment of PigF from different strains. The conserved residues are highlighted with a red background, residues (His98, His247, Asp248, Glu275 and Glu304) are represented with star.

Table S1 Strains and plasmids used in this study.

Strains and plasmids	Relevant genotype or description	References or source
**Strains**		
S. marcescens FS14	wild type strain	(Li *et al.*, 2015)
FS14ΔPigF	In-frame deletion mutant of *pigF* gene	This study
**Plasmids**		
pWDF	Suicide plasmid for construction deletion mutants	(Wu *et al.*, 2016)
pMTKQS	plasmid for complementary assay	This study
pMTKQS-*pigF*	*pigF* gene in pMTKQS	This study
pMTKQS-$pigF^{H98A}$	$pigF^{H98A}$ gene in pMTKQS	This study
pMTKQS-$pigF^{H247A}$	$pigF^{H247A}$ gene in pMTKQS	This study
pMTKQS-$pigF^{H248A}$	$pigF^{H248A}$ gene in pMTKQS	This study
pMTKQS-$pigF^{E275A}$	$pigF^{E275A}$ gene in pMTKQS	This study
pMTKQS-$pigF^{N294A}$	$pigF^{N294A}$ gene in pMTKQS	This study
pMTKQS-$pigF^{E304A}$	$pigF^{E304A}$ gene in pMTKQS	This study
pMTKQS-$pigF^{H247AD248A}$	$pigF^{H247AD248A}$ gene in pMTKQS	This study
pMTKQS-$pigF^{F148A}$	$pigF^{F148A}$ gene in pMTKQS	This study
pMTKQS-$pigF^{S298A}$	$pigF^{S298A}$ gene in pMTKQS	This study
pMTKQS-$pigF^{W131A}$	$pigF^{W131A}$ gene in pMTKQS	This study

Table S2 Primers used in this study.

Primers	Amplified regions	Sequence（5′-3′）
1	Forward primer for amplifying the upstream homologous arm of *pigF*	ATTAGGGCCCATGGGCGCCTACTTTAAGCAG
2	Reverse primer for amplifying the upstream homologous arm of *pigF*	GGCAATTTTCATGAAAAAGCCCATCATCTGATTGAC
3	Forward primer for amplifying the downstream homologous arm of *pigF*	ATGGGCTTTTTCATGAAAATTGCCGGCCCA
4	Reverse primer for amplifying the downstream homologous arm of *pigF*	CCTGGATCTCCAGCGGCG
5	Forward primer for identification of mutant FS14ΔPigF	GATCGCTTCCTGGTGCACAG
6	Forward primer for the construction of complementation plasmid of *pigF*	CGGATATAGTTCCTCCTTTCAGC
7	Reverse primer for the construction of complementation plasmid of *pigF*	AGT<u>CTCGAG</u>TTATTTTTCGCCGACGATCAGG
8	Forward primer for amplifying the downstream homologous arm of $pigF^{H98A}$	GCTGGGCCGCGCCATCGATACCTTC
9	Reverse primer for amplifying the upstream homologous arm of $pigF^{H98A}$	AGGAAGGTATCGATGGCGCGGCCCAGCCATC
10	Forward primer for amplifying the downstream homologous arm of $pigF^{H247A}$	GGATGCTGGCCGACTACG
11	Reverse primer for amplifying the upstream homologous arm of $pigF^{H247A}$	CGTAGTCGGCCAGCATCC
12	Forward primer for amplifying the downstream homologous arm of $pigF^{D248A}$	ATGCTGCACGCCTACGCCC
13	Reverse primer for amplifying the upstream homologous arm of $pigF^{D248A}$	GGGCGTAGGCGTGCAGCATC
14	Forward primer for amplifying the downstream homologous arm of $pigF^{E275A}$	TCGCTTCCGCAACGCCGC
15	Reverse primer for amplifying the upstream homologous arm of $pigF^{E275A}$	GCGGCGTTGCGGAAGCGAT
16	Forward primer for amplifying the downstream homologous arm of $pigF^{N294A}$	TGCTGTCGCGCCATGCTGGTCTCCAC
17	Reverse primer for amplifying the upstream homologous arm of $pigF^{N294A}$	GTGGAGACCAGCATGGCCAGCGACAGCAG
18	Forward primer for amplifying the downstream homologous arm of $pigF^{E304A}$	GTGGCATAGCGAGCAGCGC

19	Reverse primer for amplifying the upstream homologous arm of *pigF*^{E304A}	GCGCTGCTCGCTATGCCACC
20	Forward primer for amplifying the downstream homologous arm of *pigF*^{H247AD248A}	ATGCTGGCCGCCTACG
21	Reverse primer for amplifying the upstream homologous arm of *pigF*^{H247AD248A}	CGTAGGCGGCCAGCATC
22	Forward primer for amplifying the downstream homologous arm of *pigF*^{F148A}	ACTTCCAGGAGCGTCTCGGTAAATTCG
23	Reverse primer for amplifying the upstream homologous arm of *pigF*^{F148A}	CGAATTTACCGAGACGCTCCTGGAAG
24	Forward primer for amplifying the downstream homologous arm of *pigF*^{S298A}	GAACATGCTGGTCGCCACCGACGGTG
25	Reverse primer for amplifying the upstream homologous arm of *pigF*^{S298A}	CACCGTCGGTGGCGACCAGCATGTTC
26	Forward primer for amplifying the downstream homologous arm of *pigF*^{W131A}	ATGACCGCAGTGCGTTCGACATCCTG
27	Reverse primer for amplifying the upstream homologous arm of *pigF*^{W131A}	CAGGATGTCGAACGCACTGCGGTCAT

Underlines represent the restriction sites.

Table S3 Data-collection and structure refinement statistics.

Values in parentheses are for the highest resolution shell.

**Parameter**	apo PigF (PDB:7CLU)	PigF+SAH (PDB:7CLF)
**Data collection:**		
Wavelength (Å)	0.9796	1.0088
Temperature (K)	100	100
Crystal-to-detector distance (mm)	220	250
Rotation range per image (°)	1.0	1.0
Total rotation range (°)	180	180
Space group	$P12_11$	$P12_11$
Unit-cell parameters (Å)	a = 69.10,<br>b = 52.36,<br>c = 92.83,<br>β = 97.35	a = 43.9,<br>b = 109.2,<br>c = 63.9,<br>β = 93.1
Resolution range (Å)	19.57–1.90 (1.94–1.90)	19.65–1.97 (2.02–1.97)
Observed reflections	105469 (7133)	159034 (11129)
Unique reflections	48618 (3366)	42182 (2963)
Multiplicity	2.2 (2.1)	3.8 (3.8)
Rpim (%)	0.052 (0.624)	0.078(0.567)
Completeness (%)	93.6 (96.5)	99.6 (99.8)
$\langle I / \delta(I) \rangle$	7.9 (1.2)	9.9 (1.5)
CC (1/2)	0.996 (0.506)	0.995 (0.628)
**Structure refinement**		
Total number of atoms	5273	5682
Number of reflections used	48590	41496
R work (%)	19.87	18.53
R free (%)	25.06	25.34
RMSD bonds (Å)	0.01	0.007
RMSD angles (°)	0.85	0.891
**Ramachandran plot (%)**		
Favoured	98.42	98.96
Allowed	1.42	1.04
Outlier	0.16	0

Table S4 DALI Structural Similarity Search.

PDB ID	Z-score[a]	RMSD[b]	Residues	Indentity%	Protein	Reference
**4A6D**	32.9	2.7	346	27	Human N-acetylserotonin methyltransferase ASMT	Ref. (Botros *et al.*, 2013)
**3GWZ**	30.8	2.5	340	23	*Streptomyces lavendulae*: mitomycin-7-O-methyltransferase MmcR	Ref. (Singh *et al.*, 2011)
**1TW3**	30.6	2.7	340	23	*Streptomyces peucetius*: carminomycin 4-O-methyltransferase DnrK	Ref. (Jansson *et al.*, 2004)
**3I58**	30.0	2.9	329	21	*Streptomyces carzinostaticus*: neocarzinostatin O-methyltransferase NcsB1	Ref. (Cooke *et al.*, 2009)
**1XDS**	28.6	3.0	336	24	Aclacinomycin 10-Hydroxylase RdmB	Ref. (Jansson *et al.*, 2005)
**1FP2**	27.9	3.7	346	18	Medicago sativa: isoflavone O-methyltransferase IOMT	Ref. (Zubieta *et al.*, 2001)
**3P9I**	27.7	3.7	358	23	Perennial Ryegrass: caffeic acid O-methyltransferase LpOMT1	Ref. (Louie *et al.*, 2010)
**2IP2**	27.6	3.8	330	20	*Pseudomonas aeruginosa*: pyocyanin biosynthetic protein PhzM	Ref. (Parsons *et al.*, 2007)
**1KYW**	27.4	3.6	361	22	Alfalfa: caffeic acid/5-hydroxyferulic acid 3/5-O-methyltransferase COMT	Ref. (Zubieta *et al.*, 2002)
**4EVI**	27.0	3.4	361	20	*Linum nodiflorum*: coniferyl alcohol 9-O-methyltransferase Ca9OMT	Ref. (Wolters *et al.*, 2013)
**3LST**	26.6	3.1	334	21	Orsellinic acid C3 O-methyltransferase CalO1	Ref. (Chang *et al.*, 2011)
**1FP1**	25.6	4.3	341	22	Medicago sativa: chalcone O-methyltransferase ChOMT	Ref. (Zubieta *et al.*, 2001)

[a] Z-score represents the statistical significance of the best domain-domain alignment.

[b] RMSD represents the root mean square deviation of C atoms in rigid body superimposition.
